# Orientational Mapping Augmented Sub-Wavelength Hyper-Spectral Imaging of Silk

**DOI:** 10.1038/s41598-017-07502-3

**Published:** 2017-08-07

**Authors:** Meguya Ryu, Armandas Balčytis, Xuewen Wang, Jitraporn Vongsvivut, Yuta Hikima, Jingliang Li, Mark J. Tobin, Saulius Juodkazis, Junko Morikawa

**Affiliations:** 1 Tokyo Institute of Technology, Meguro-ku Tokyo, 152-8550 Japan; 2 Nanotechnology facility, Center for Micro-Photonics, Swinburne University of Technology, John st., Hawthorn, Victoria 3122 Australia; 3 Department of Laser Technologies, Center for Physical Sciences and Technology, Savanoriu Ave. 231, LT-02300 Vilnius, Lithuania; 4 Infrared Microspectroscopy Beamline, Australian Synchrotron, Clayton, Victoria 3168 Australia; 5 Department of Chemical Engineering, Graduate School of Engineering, Kyoto University, Nishikyo-ku, Kyoto 615-8510 Japan; 6 Institute for Frontier Materials, Deakin University, Waurn Ponds, Victoria, 3217 Australia; 7 Center of Nanotechnology, King Abdulaziz University, Jeddah, 21589 Saudi Arabia; 8 Melbourne Center for Nanofabrication, Australian National Fabrication Facility, Clayton, 3168 Australia

## Abstract

Molecular alignment underpins optical, mechanical, and thermal properties of materials, however, its direct measurement from volumes with micrometer dimensions is not accessible, especially, for structurally complex bio-materials. How the molecular alignment is linked to extraordinary properties of silk and its amorphous-crystalline composition has to be accessed by a direct measurement from a single silk fiber. Here, we show orientation mapping of the internal silk fiber structure via polarisation-dependent IR absorbance at high spatial resolution of 4.2 *μ*m and 1.9 *μ*m in a hyper-spectral IR imaging by attenuated total reflection using synchrotron radiation in the spectral fingerprint region around 6 *μ*m wavelength. Free-standing longitudinal micro-slices of silk fibers, thinner than the fiber cross section, were prepared by microtome for the four polarization method to directly measure the orientational sensitivity of absorbance in the molecular fingerprint spectral window of the amide bands of *β*-sheet polypeptides of silk. Microtomed lateral slices of silk fibers, which may avoid possible artefacts that affect spectroscopic measurements with fibers of an elliptical cross sections were used in the study. Amorphisation of silk by ultra-short laser single-pulse exposure is demonstrated.

## Introduction

The infra-red (IR) spectral region from 3–10 *μ*m, referred to as the fingerprint region, is used for the quantitative analysis of molecular species in a wide range of applications spanning fields of climate change^1^, environmental monitoring^2^, bio-medical^3^, material science^4^, and security^5^. All imaging methods have mounting challenges to characterise volumes with cross sections approaching the wavelength of the utilised light. In the UV-visible and IR spectral domains, near-field techniques using sharp nano-tips and plasmonic enhancement are used to reach nanoscale spatial resolutions, usually at the expense of polarisation information. However the application of polarised light permits analysis of the molecular orientation and chirality, which define mechanical, thermal, and optical properties^6^. At different wavelengths it is possible to access orientational information of hierarchical structures which underpins mechanical material properties and could be harnessed by engineering their artificial counterparts^4^.

Fourier transform IR (FT-IR) spectroscopy, when combined with a microscope accessory, provides hyper-spectral imaging when spectrally broadband or wavelength-tunable excitation sources are utilised. In the IR spectral range, a combination of sub-wavelength spatial resolution to characterise the anisotropy of absorbance due to local molecular orientation and spatial 2D (3D) mapping would enhance current analytical techniques and has high potential in material and bio-medical fields. In addition the use of a synchrotron beam offers highly collimated IR radiation with 10^2^–10^3^ times higher brightness than that available from laboratory-based IR sources (Globar^®^). Such a unique characteristic enables the acquisition of high-quality FT-IR spectra at diffraction-limited spatial resolution, making synchrotron-IR microspectrscopy an excellent analytical platform for acquiring spatially resolved chemical images of materials at a lateral resolution between 3–10 *μ*m. Using attenuated total reflection (ATR) with a high refractive index *n* = 4 Ge contact lens, a state-of-the-art resolution of 1.9 *μ*m, which is sub-wavelength in the IR molecular finger printing spectral range, can be achieved and was one of the aims of this study.

The field of bio-medical applications could be one of the main beneficiaries of high-spatial resolution techniques with a focus on sensors and bio-materials. In protein based materials, the molecular ordering, orientation, and conformation define their properties^6^. Silk was the material of choice in this study due to its bio-compatibility and bio- degradability^7,8^. It has high mechanical strength with rich structural and compositional complexity ranging from *α*-coils (IR absorbance at 1660 cm^−1^), metastable *β*-turns (silk I), crystalline *β*-sheets (silk II), and amorphous random fibroin protein structure^9^. Controlled modification of silk structure from water soluble amorphous phase to crystalline *β*-sheets is a current focus of research^10–12^, with structural characterisation of silk having been carried out with X-ray diffraction (XRD), nuclear magnetic resonance (NMR), and IR spectroscopy of silk fiber bundles and amorphous powders^13,14^.

A systematic study on orientational properties of the building blocks of the crystalline-amorphous hierarchial structure of single silk fibers which is essential to understanding the properties of silk, e.g., why a faster reeling makes stronger fibers^15,16^ and how it is linked to fragility and relaxation in polymers^17^ is highly required. Structure of single spider silk fibers was investigated by XRD including changes due to water uptake^18,19^. Differences of spectral band positions using free space IR and ATR-IR spectroscopies^20,21^ and order parameter determination^22,23^ have been carried out for single fibers. Synchrotron X-ray microscopy was used to reveal orientational effects in absorbance of spider silk at high spatial resolution ~50 nm^23,24^. A polarisation dependence of the IR absorbance of amides in silk fiber can provide deeper insights in molecular orientation of hierarchial silk structure; it is known to define thermal conductivity, *κ*, which is increasing in the stretched form $\kappa \sim \sqrt{E}$^25^ (*E* is the Young’s modulus) and is increasing under strain towards the onset of melting at around 200 °C^26^. In the presence of hydrogen bonding, the orientation is linked to an increased crystallinity^27,28^. Nanoscale orientation of proteins and their 3D conformation are at the core of their optical, mechanical, thermal, and bio-functions. These important properties can be better understood using high resolution techniques, which have to be applied simultaneously for space and spectrum measurements to unveil primary and secondary molecular orientation/alignment. The polarisation dependence of the absorbance bands is used to determine anisotropy of absorbance in silk. It allows to investigate structure of silk at nanoscale^22^ and relate it to the hierarchical structure and mechanical properties^29–31^.

Here, sub-wavelength spatial resolution was combined with hyper-spectral imaging to characterise local absorbance of silk fibers modified by ultra-short laser pulses using the in-house developed ATR FT-IR instrument at Australian Synchrotron. Polarisation dependence of the absorbance was successfully invoked to reveal the high degree of orientation of amide building blocks of silk in fibers and to recognise laser-induced amorphisation. In order to exclude shape related effects in absorbance measurements and to reveal molecular orientation along the silk fiber, thin and flat microtome slices of lateral silk fiber were prepared and used in this study.

## Experimental

**Figure 1:**
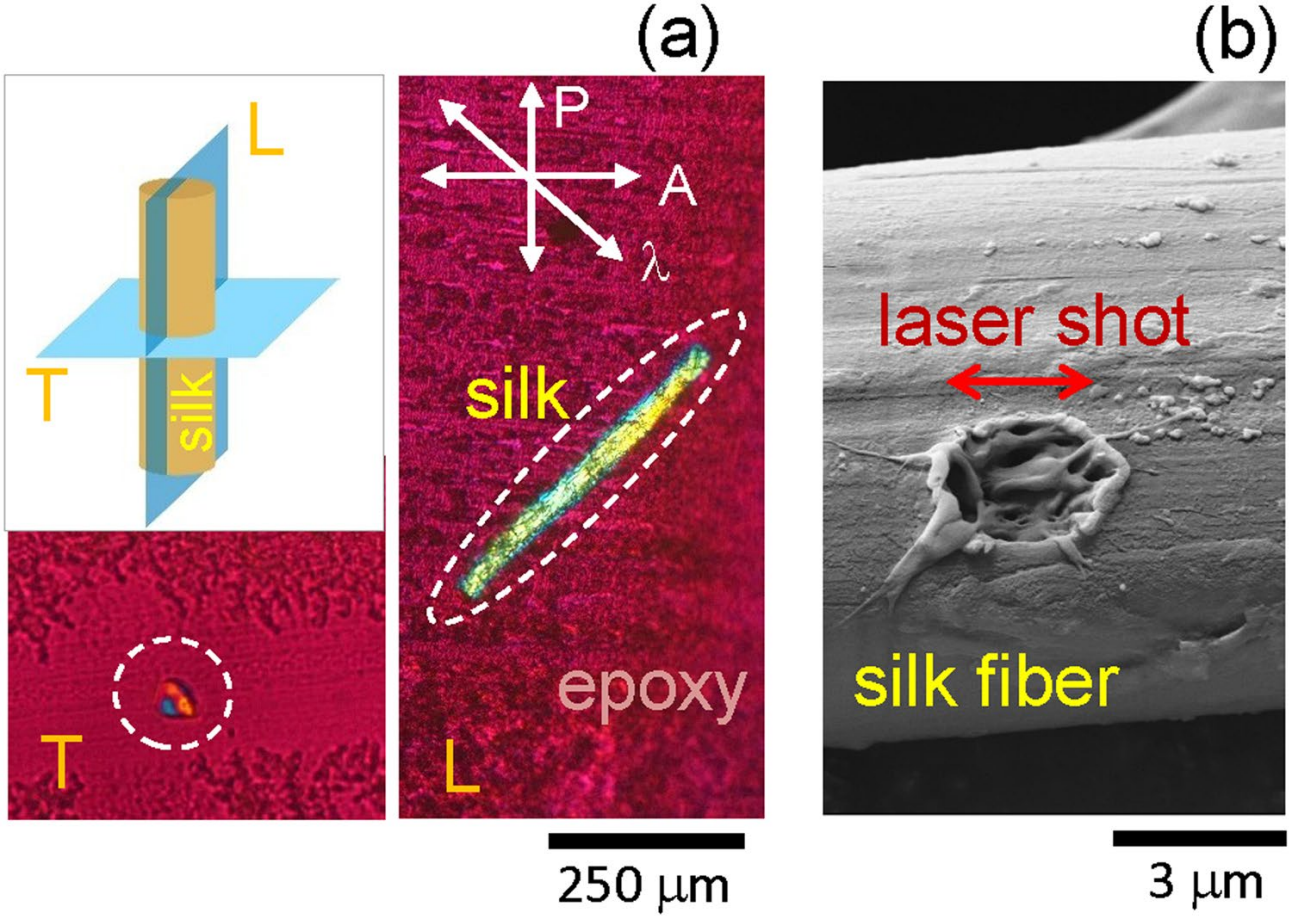
(**a**) Longitudinal (L) and transverse (T) 5–10 *μ*m-thin slices of silk embedded in epoxy were used for FT-IR micro-spectroscopic characterisation. Cross-polarised optical image of the sliced silk fiber obtained with a waveplate (*λ*) shifter. Sample preparation: silk fiber was aligned and epoxy embedded, microtome sliced for L and T directions. (**b**) SEM image of a single laser pulse melted silk; laser wavelength 515 nm, pulse duration 230 fs, focused with objective lens with numerical aperture *NA* = 0.5, pulse energy 85 nJ, linear polarisation was along the fiber (marked by arrow).

**Figure 2:**
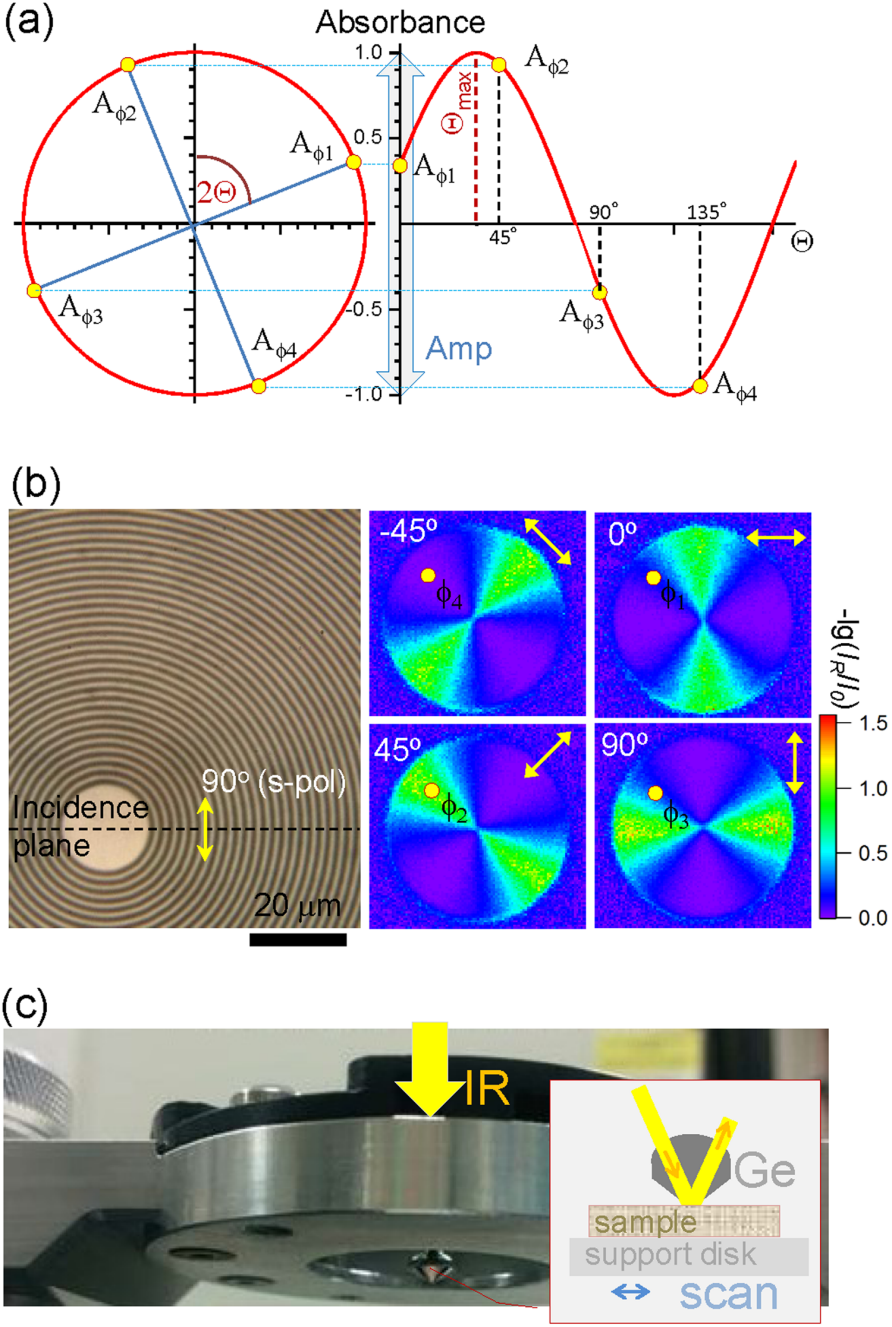
(**a**) Four-polarisation method in IR absorbance at different polarisation azimuths, *A* = *f*(**Θ**)^32^, which was used to determine the orientational anisotropy in far-field FT-IR measurements. (**b**) An optical image of a concentric 2-*μ*m-period Au grating (with a duty cycle of 0.5) on a cover glass used for reflection FT-IR imaging carried out with a Cassegrainian objective lens with a focusing cone over a 17-to −30° range; dashed-line marks the top-view of the incidence plane. The diameter of the circular grating was 0.5 mm. Four reflection maps for a linearly polarised incidence at the specified polarisation angle (schematically also marked by arrows) at 1500 cm^−1^ (~6.7 *μ*m) wavelength (Spotlight, PerkinElmer); *I*_*R*,0_ are the reflected and incidence intensities, respectively. (**c**) In-house developed ATR accessory for Hyperion 2000, Bruker microscope. Inset shows schematic illustration of optical geometry used in the ATR FT-IR measurement with a 100-*μ*m diameter Ge tip (refractive index *n* = 4).

Silk samples were cut to a thickness of a few micrometers by microtome (Fig. 1), then laser modified by single laser shots before FT-IR measurements at the IR Microspectroscopy Beamline (Australian Synchrotron) using a polarisation discrimination method for the far-field absorbance measurement^32^ and subsequently at a high spatial resolution using in-house developed ATR accessory (Fig. 2).

### Silk micro-slices

Domestic silk (*Bombyx mori*) fibers were used for experiments after removal of sericin rich cladding^12^. For the cross-sectional observation, the natural silk fibers were aligned and embedded into an epoxy adhesive (jER 828, Mitsubishi Chemical Co., Ltd.). Fibers fixed in the epoxy matrix were cut in 1–5 *μ*m-thick slices which were found to possess sufficient mechanical robustness for the FT-IR transmission measurements carried out without any supporting substrate. This was important to increase sensitivity of the far-field absorbance measurements and to decrease reflective losses that may occur through use of a supporting substrate. Longitudinal (L) and transverse (T) slicing of the silk fibers was carried out by microtome (RV-240, Yamato Khoki Industrial Co., Ltd.; see Fig. 1). The slices, which were cut thinner than the original silk fibers, were used for the transmission measurements in mapping mode along and across the fiber without background interference from a supporting epoxy host. For the ATR FT-IR, an aluminium disk was used to mount the thin fiber cross section, which was subsequently brought into contact with a 100-*μ*m-diameter sensing facet of the Ge ATR hemisphere (refractive index *n* = 4).

Modification of silk was carried out using 515 nm wavelength and 230 fs duration pulses (Pharos, Light Converison Ltd.) focused with an objective lens of numerical aperture *NA* = 0.5 (Mitutoyo). Single pulse modifcations were carried out with pulse energy, *E*_*P*_, indicated at the irradiation point, using an integrated industrial laser fabrication setup (Workshop of Photonics, Ltd.). Optical and scanning electron microscopy (SEM) were used for structural characterisation of the laser modified regions.

### Four-polarisation method

Anisotropy of the far-field absorbance can be quantified using the four polarisation method^32^ by measuring absorbance at four polarisations separated by a *π*/4 azimuth and assuming a linear absorption of molecular dipoles in the E-field of light. A sine wave profile of absorbance fit is expected (Fig. 2a) with the min-max amplitude of absorbance, *Amp*, and dipole orientation angle, *θ* defined for each pixel of a hyper-spectral image^32^:

1 $$Amp=\sqrt{{({A}_{{\phi }_{4}}-{A}_{{\phi }_{2}})}^{2}+{({A}_{{\phi }_{3}}-{A}_{{\phi }_{1}})}^{2}},$$

2 $${\theta }=\frac{1}{2}{\tan }^{-1}(\frac{{A}_{{\phi }_{3}}-{A}_{{\phi }_{1}}}{{A}_{{\phi }_{4}}-{A}_{{\phi }_{2}}}),$$

where ${A}_{{\phi }_{1,2,3,4}}$ are absorbance at the four polarisation azimuths separated by π/4; *Amp* = *A*_*max*_ − *A*_*min*_ is defined by the maximum and minimum absorbances.

This four-polarisation method was implemented using a Cassegrainian FT-IR objective with the linear polarisation set right at the entrance of the objective lens by a wire-grid polariser. To test the validity of the four-polarisation method for this geometry, where two reflections on curved mirrors are encountered by linearly polarised incident beam in the Cassegrainian optic, a circular grating reference sample was made by electron beam lithography (EBL; ACE-7000/EBU, Sanyu Electron Ltd.) and a standard lift-off method. A 30 nm-thick Au coating was thermally evaporated on a 10 nm adhesion layer of Cr on a cover glass for the lift-off over the EBL defined circular pattern in ZEP520 resist; diameter of the circular grating was 0.5 mm. The grating with a width of Au rings of 1 *μ*m and period of 2 *μ*m represents a reflective sub-wavelength pattern of a constantly changing orientation at the IR wavelength of 1500 cm^−1^ or ~6.7 *μ*m (Fig. 2b). By setting four polarisations with a *π*/4 separation at incidence, the reflection maps from the circular grating measured with Spotlight, PerkinElmer are shown in Fig. 2b. Angular integration of the reflected intensity at any radial position closely followed the postulated sine wave rule (Fig. 2a); e.g., the four selected angle positions on the reflection maps are marked by *ϕ*_1,2,3,4_ and follow intensity changes by the sine wave form. The strongest reflection was observed for the polarisation which is tangential to the circumference of the grating ring pattern.

### High spatial resolution FT-IR spectroscopy

The far-field transmission measurements were carried out with a *NA* = 0.5 and 36× magnification Cassegrainian objective lens. A wire-grid ZnSe polariser was used to set linear polarisation (Specac Ltd., Kent, UK).

Synchrontron IR microspectroscopic measurement was performed using a Bruker Hyperion 2000 FT-IR microscope (Bruker Optik GmbH, Ettlingen, Germany) coupled to a Vertex V80v FT-IR spectrometer, and equipped with a liquid nitrogen-cooled narrow-band mercury cadmium telluride (MCT) detector. As illustrated in Fig. 2c the in-house developed ATR FT-IR accessory equipped with a 100-*μ*m-diameter facet Ge ATR crystal was used to acquire chemical images of the silk cross sections at a high speed and a spatial resolution down to 1.9 *μ*m^33^. The Ge contact lens of $NA=nsin\psi \simeq 2.4$ was used with *n* = 4 and the *ψ* = 36.9° half-angle of the focusing cone. Deep sub-wavelength resolution $r=0.61{\lambda }_{IR}/NA\simeq \mathrm{1.5\ }\mu$m is achievable for the IR wavelengths of interest at the amide band of *λ*_*IR*_ = 1600 − 1700 cm^−1^ or 6.25–5.9 *μ*m. Use of the solid immersion lens also leads to a reduction of the mapping step size by ~4 times relative to the stage step motion and was 250 nm.

## Results

### Polarisation dependence at single point

**Figure 3:**
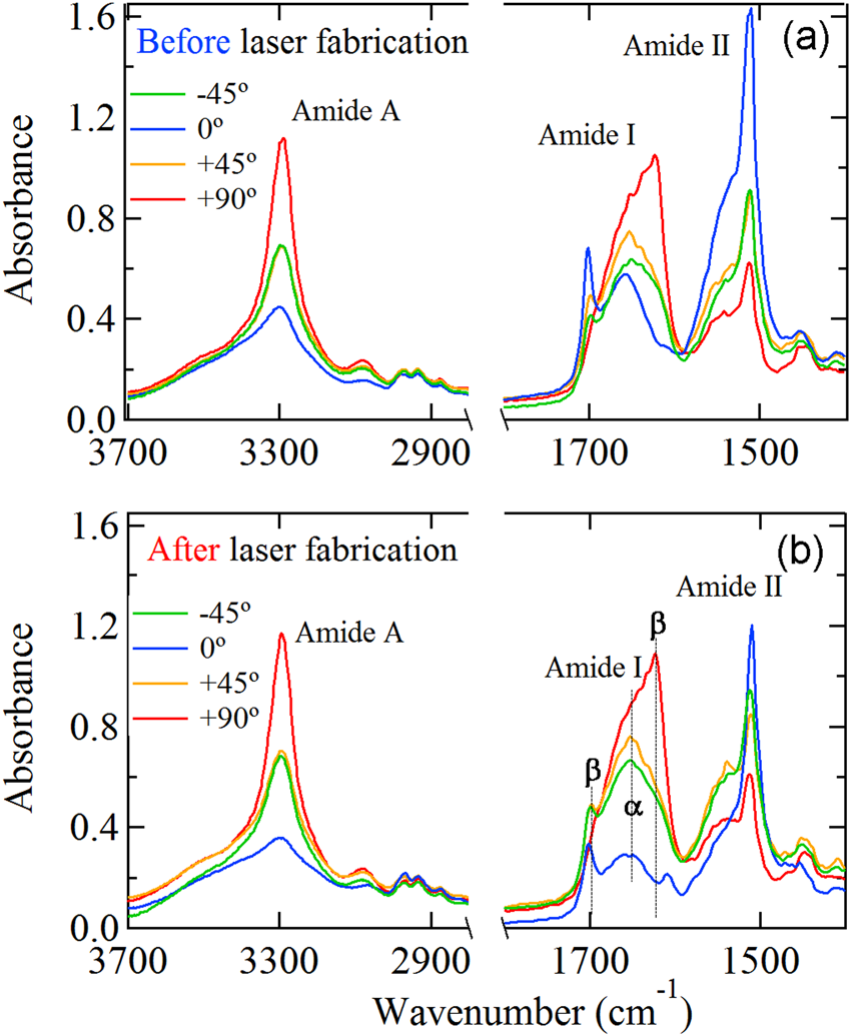
Polarisation discriminated absorbance spectra of pristine (**a**) and laser 515 nm/230 fs irradiated (**b**) silk fiber; laser pulse energy was 8.5 nJ and pulse-to-pulse separation of 2 *μ*m in xy-array. Area of laser patterning was 10 × 10 *μ*m^2^; IR beam diameter at focus on the sample was 4.2 *μ*m.

Figure 3 shows absorbance of silk measured in transmission for four different azimuthal orientations of the linear polarisation with an angular separation of *π*/4 for silk (*Bombyx mori*) from laser exposed (a) and un- treated (b) regions. A xy-array of laser irradiated spots at 8.5 nJ/pulse was patterned with 2*μ*m period while the IR spectra were acquired from a 4.2 *μ*m spot. The Amide I and II regions^34^ were investigated for structural and compositional changes induced by laser irradiation. The Amide II band at 1508 cm^−1^ is assigned to *β*-sheet secondary structure, whilst the peak at 1546 cm^−1^ is associated with disordered (amorphous) fibroin. The Amide I band follows a similar distribution with components at ~ 1625 cm^−1^ (*β*-sheets) and 1648 cm^−1^, which are associated with irregular structures including random coil and extended chains^34^. Other characteristic bands are associated with Silk I, type II *β*-turns (1647–1654 cm^−1^), *α*-coils (1658–1664 cm^−1^) as well as turns and bends 1699 cm^−1^^35^.

Laser irradiation was found to strongly affect the sharp absorbance peak at 1700 cm^−1^ when laser pulse energy was exceeding the threshold of structural damage at ${E}_{p}\simeq 8$ nJ for the used focusing (Fig. 3). This is indicative of amorphisation, which would be expected based on the observed changes in the SEM images of the laser exposed silk shown in Fig. 1b. A distinct polarisation dependence was also observed, as expected from a crystalline rich (~85%) silk fibers at the *β*-sheet Amide I band. The strongest absorbance at the Amide II (C-N) stretching band at 0° polarisation corresponded to the C-N bond, which is aligned along the fiber direction. The Amide I (C=O) stretching band, on the other hand, showed an inverse correlation with the polarised absorbance spectrum of the Amide II band which was strongest at the perpendicular polarisation (Fig. 3) as expected from previous Raman scattering studies^36^. The N-H stretching band showed the same polarisation dependence as the C=O stretching band. Due to such unique and strong polarisation dependence of the absorbance at a single spot (Fig. 3), the far-field transmission measurement in the mapping mode was subsequently performed to gain additional insights into molecular orientation/alignment along the length of silk fibers made accessible via microtomed L cross sections.

### Molecular orientation in silk: far-field case

**Figure 4:**
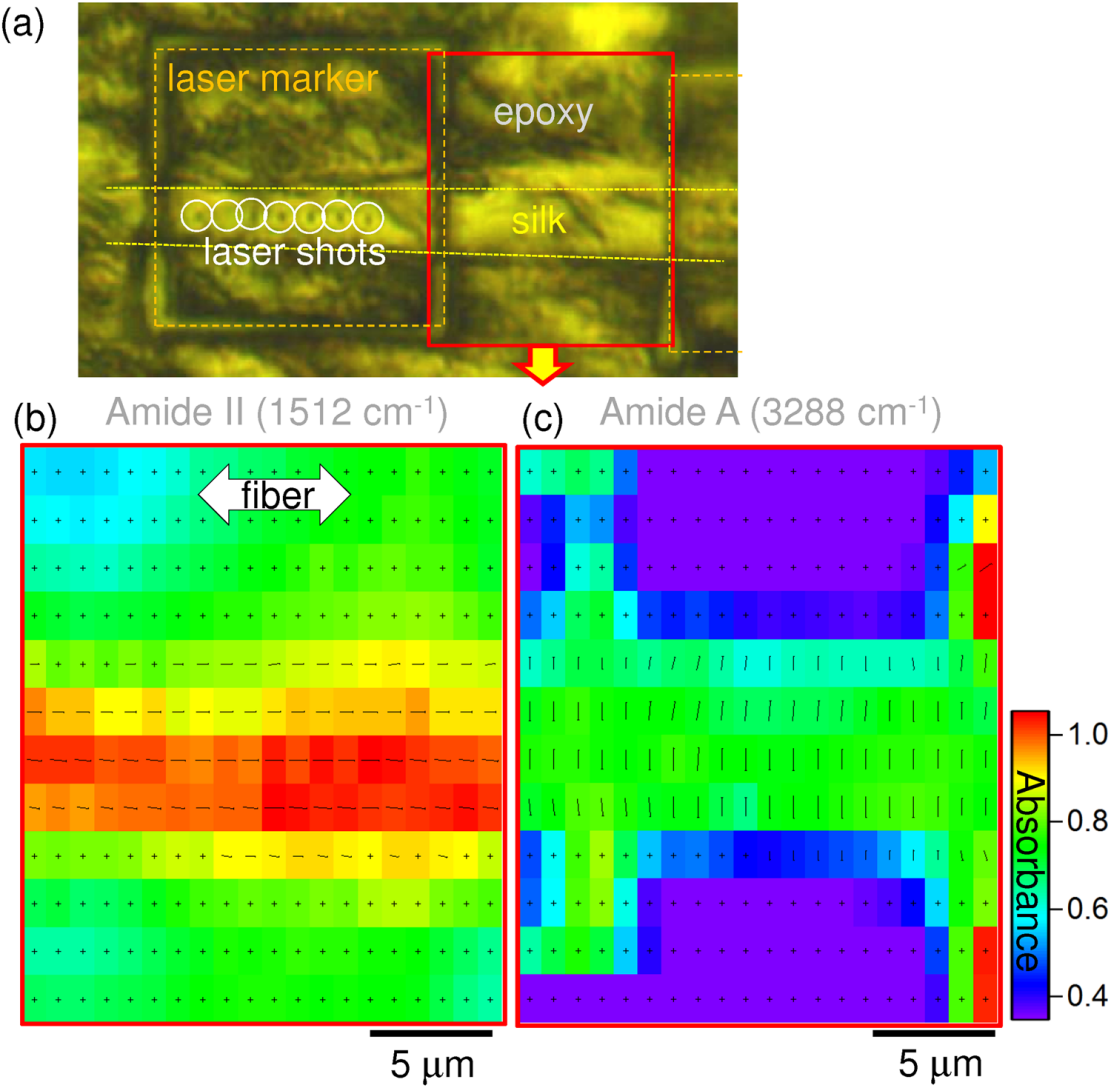
(**a**) An optical image of the L cross section of a *Bombyx mori* silk fiber with laser marked 20 × 20 *μ*m^2^ regions and laser irradiated spots. The region mapped in (**b**,**c**) is shown in a solid rectangle in (**a**). (**b**,**c**) Orientation vector map (marker’s length Eqn. 1 and orientation Eqn. 2) overlayed with the far-field absorbance (color map) at the C-N (**b**) and N-H (**c**) bands; these bonds are known to be perpendicular. S-polarised incident light was perpendicular to the fiber; in the plane of image.

The four polarisations method was applied to reveal orientational association of the amide bands using Eqns. 1 and 2. Figure 4 shows the chemical maps of the L cross section of silk fiber with measured ~4.2*μ*m spatial resolution (*NA* = 0.5). Mapping data (as measured) are visualised by overlaying absorbance at the selected wavenumber values of Amide II and Amide A at 1512 cm^−1^ and 3288 cm^−1^, respectively. The corresponding vector plot (markers’ length Eqn. 1 and orientation Eqn. 2) revealed that the orientation is horizontal and the amplitude *Amp* is proportional to the length of the bar-marker (*θ* = 0° is horizontal). The vector plot represents a background-free component of absorbance change caused by a change in molecular alignment. Perpendicular orientation between C=O and C-N bonds observed in the single spot spectrum (Fig. 3) has been confirmed for the non-irradiated silk regions (Fig. 4(b) vs (c)). Nevertheless, some of the Amide II bands present in the epoxy matrix were found to possess a random orientation.

To quantify the order the standard second momentum *P*_2_(θ) of the orientation function also known as the Herman’s function can be expressed via the absorbance ratio at two perpendicular linear polarisations, the dichroic ratio, $D={A}_{{0}^{^\circ }}/{A}_{{90}^{^\circ }}$ (see Supplement for details) as^32,37^:

3 $${P}_{2}(\theta )=\frac{3\langle {\cos }^{2}\theta \rangle -1}{2},$$

where *θ* is the angle between the transition dipole moment and the selected orientation (along silk fiber). The second momentum of the C=O (Amide I) band was found *P*_2_(*θ*) = −0.36 from the Raman measurements^36^ (−0.5 corresponds to a pure perpendicular orientation to the fiber axis). A slightly less ordered C=O bonds were determined in this study with *P*_2_(*θ*) = −0.29 ± 0.026 (see, Supplement for details). The difference can be accounted by a fiber caused anisotropic diffraction in the case of Raman measurements while a flat cross section was used in this study. The order analysis reveals that silk fibers are up to ~60% crystalline (see, Supplement) which is approximately twice larger than observed by synchrotron FT-IR in regenerated silk fibroin after crystallisation in alcohol bath ~28%^38^.

### High resolution ATR mapping

**Figure 5:**
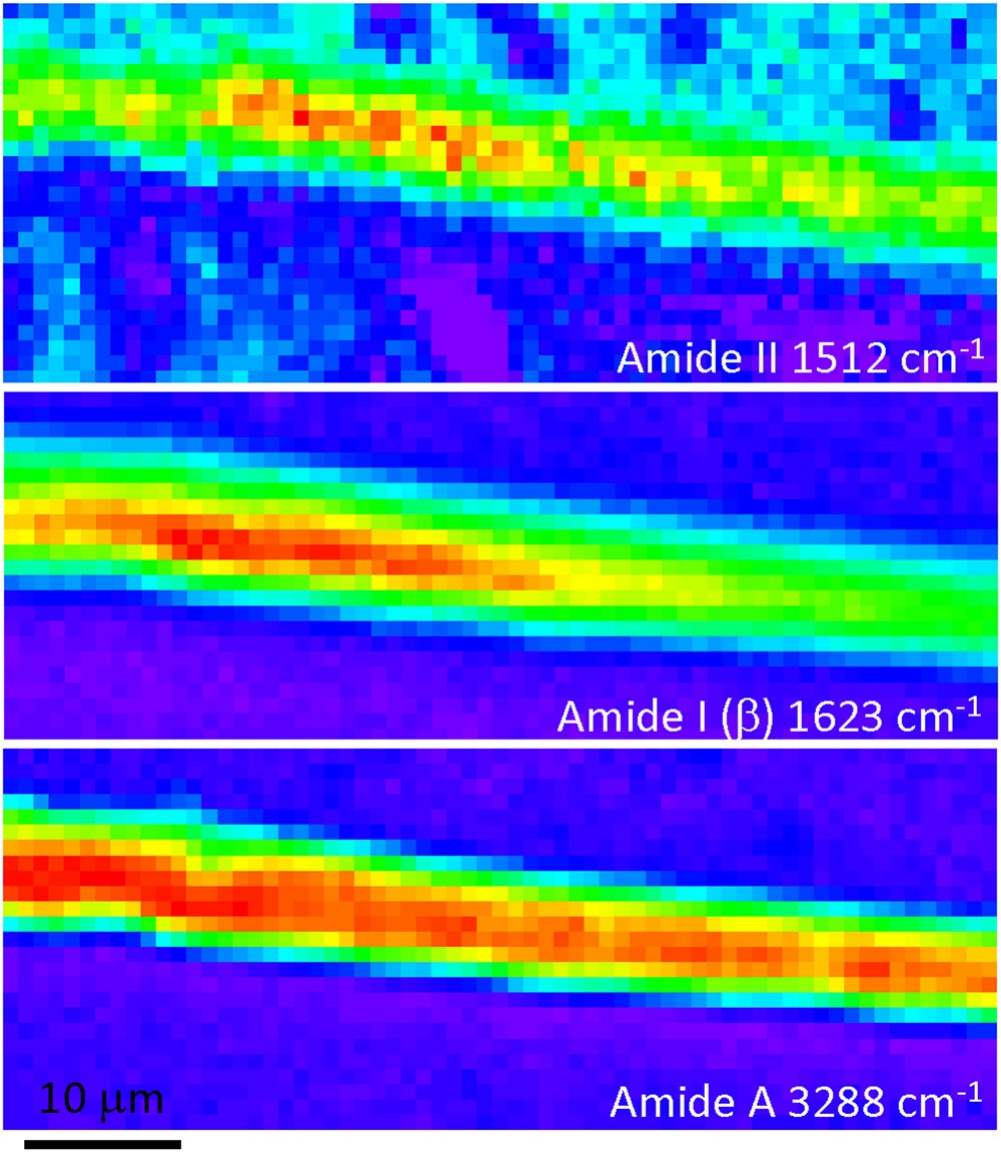
High resolution 1.9 *μ*m ATR FT-IR maps at 1.9 *μ*m resolution of the longitudinal (L) cross sections of silk presented in auto-scale for better viewing; a background-corrected absorbance is ranging from 0 to approximately 0.2. Lateral step size between pixels was 0.5 *μ*m; as-measured pixelated absorbance maps are presented. Polarisation of incident light onto ATR prism was *s* (in the plane of image; along y-axis).

The highest spatial resolution was achieved using ATR FT-IR, with a *NA* = 2.6 focus, realised using the combination of a germanium solid immersion lens of *n* = 4, with a Cassegrainian objective of *NA* = 0.6 and 20× magnifcation. Although no polariser was used for the mapping, synchrotron beam had a dominant s-polarised linear component. It should be emphasised, that in addition to the enhanced lateral spatial resolution this ATR FT-IR optical configuration offered high surface sensitivity due to a low penetration depth of 0.5 *μ*m of the IR radiation at the amide I absorption peak. Figure 5 shows the highest spatial resolution $r\simeq \mathrm{1.9\ }\mu$m chemical maps of the L section of silk at a few spectral regions of interest selected from a single hyper- spectral FT-IR data set. The Amide A (N-H) band appeared to have the most uniform distribution across the fiber compared to those of the Amide I (C=O) and *β*- sheet, which were highly localised inside the core of the fiber. This could be rationalised by the low sensitivity of N-H absorption to a surrounding hierarchial structure of the protein matrix, mainly, because of a low mass of hydrogen.

**Figure 6:**
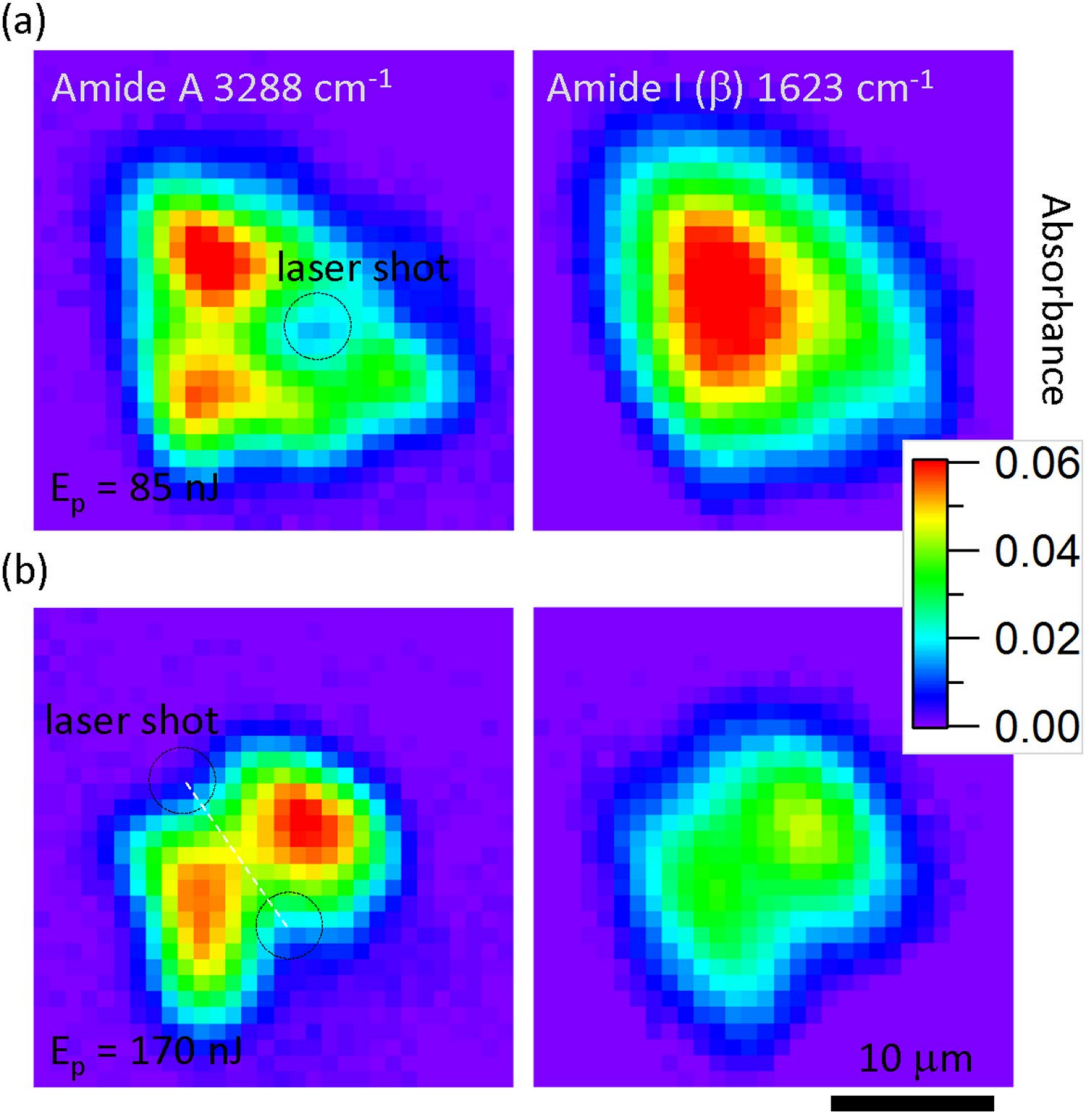
High resolution 1.9 *μ*m ATR maps of transverse (T) cross sections of silk (*Bombyx mori*) with laser 515 nm/230 fs irradiated spots; laser pulse energies, *E*_*p*_ = 85 nJ (**a**) 170 nJ (**b**) on the sample; the polarisation was linear. The lateral step size was 0.5 *μ*m; as-measured pixelated absorbance maps are presented. Polarisation of incident light onto ATR prism was *s* (in the plane of image; along y-axis).

The distinct effect of laser irradiation on the molecular structure of silk fibers was revealed in this study for the first time by high-spatial resolution ATR FT-IR mapping of T-cross sections (Fig. 6). Single laser shots created an approximately 2 *μ*m diameter ablation pits observable through both optical or/and SEM images (Fig. 4a). The pattern of irradiation spots was controlled with a high precision of ~50 nm. This was instrumental in identifying the irradiation locations on the absorbance maps (Fig. 6). Central localisation of *β*-sheets is well distinct in the T sectional images (Fig. 6).

The lowest laser pulse energy which made recognisable modifications to the fibres in a single shot was $E\simeq 8$ nJ. Spectral maps in Fig. 6a show that only modification at the Amide A band was observed after laser irradiation, while Amide I *β*-sheet structure was not affected. By doubling the laser pulse energy, the distribution of both the Amide A and I bands were found altered (Fig. 6b). This finding is consistent with the chemical bond strength which are 189 kJ/mol (Amide A) and 1076.5 kJ/mol (Amide I) in *β*-sheet, respectively^40^.

## Discussion

Substrate-free absorbance measurements of silk fibers, with lateral resolution defined by *NA* = 0.5 for the far-field transmission and *NA* = 2.4 for the ATR FT-IR hyper-spectral mapping, have shown consistency between spatial localisation of the Amide I and II bands in the silk fiber. The fiber core is *β*-sheet enriched, hence, crystalline, as revealed by L and T cross sections of silk fibers. Flat microtome slices eliminated fiber shape related optical distortions and allowed measurements of order parameters of the amide bands (see, online supplement). Such L-cross sections can be also beneficial for determination of order parameters by Raman scattering.

**Figure 7:**
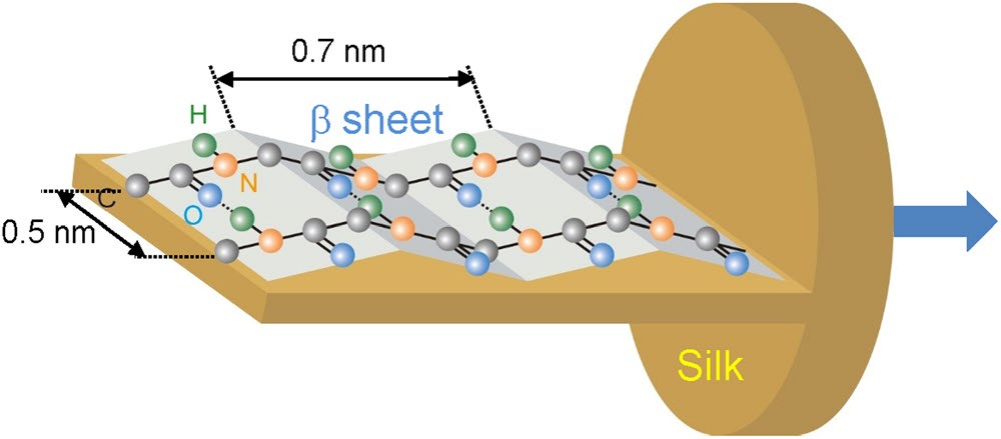
The orientation of the C = O, C-N, and N-H bonds in amide structure of the L-section of silk fiber^23,39^ confirmed in this study by the hyper-spectral imaging (see, Fig. 4). Only the in-plane components of *β*-sheets are drawn without out-off-plane alkyl moieties; hydrogen bonding responsible for *β*-sheet crystallisation is shown by the dotted line O $\cdots$ H. An arrow marks fiber drawing (strain) direction important to alignment of *β*-sheets. Microtome slices allowed to measure absorbance of the lateral flat cross sections without introduction of a fiber shape related anisotropy.

The four polarisation method was adopted in transmission mode for the high-brightness synchrotron IR radiation and applied to the L section of silk fibers to reveal unambiguously the orientational structure of the amide bands as illustrated in Fig. 7. The Amide A (N-H) and amide I (C=O) have slightly different *P*_2_ order parameters. It was confirmed that Amide A and Amide II bands are perpendicular (see, online supplement). The spatial mapping functionality demonstrated in this study possesses a capability to reveal silk amorphisation mechanisms activated by application of tightly focused ultra-short laser pulses. This distinct laser irradiation is required for a fast thermal quenching in excess of 2 × 10^3^ K/s for solidification of amorphous silk melts^11^. Understanding the mechanisms of amorphous fibroin crystallisation induced by ultra-short laser pulses at the ablation threshold of glass substrate^12^ requires structural sensitivity at high spatial resolution to confirm the role of electron avalanche in the formation of crystalline *β*-sheets in direct laser printing^41^. The 3D laser printing of silk scaffolds by a direct write approach has a strong potential for bio-medical implants, e.g., a plasma laser deposition of crystalline silk^42^ and *β*-sheet formation form amorphous fibroin under 266 nm laser irradiation^43^ have been demonstrated. By applying stretching to films of pure sericin, which is amorphous in silk fiber cladding, a molecular orientation can be imprinted^44^. Cast-drying of volumetric silk workpieces for a mechanical post-processing in orthopedic applications has been recently demonstrated with a need to control nano-/micro-structure for the required specific strength and modulus (stiffness)^45–49^ which can also be controlled by molecular alignment.

## Conclusions

High spatial resolution has been achieved in hyper-spectral imaging ATR FT-IR imaging as demonstrated by the ~1.9*μ*m (*NA* = 2.4) resolution chemical imaging of silk at ${\lambda }_{IR}\simeq \mathrm{6\ }\mu$m wavelengths. It is shown that the four polarisation method can be effectively used to reveal a prevalent orientational ordering using far-field IR absorbance mapping. In silk, a strong correlation between orthogonal dipole transition of C=O and C-N bonds has been confirmed. The order parameters of the amides was determined using micro-thin flat longitudinal microtome slices. For the C=O, order parameter *P*_2_(*θ*) = −0.30 ± 0.04 is comparable with values obtained by different methods^22,23,36^. This four polarisation method can be used to recognise laser induced amorphisation of silk. Amorphous silk is water soluble.

Insights into the orientational structure of biomaterials responsible for their optical, mechanical, and thermal properties is critical for applications and design of new materials. Here a direct absorbance measurement of orientation of the chemical bonding in silk at a record high spatial resolution is reported using synchrotron based ATR FT-IR microspectroscopy. This technique has been shown to possess potential as a powerful analytical tool for a wide range of applications capable of establishing the link between micro-/nano-structures and specific properties of biomaterials. Hyper-spectral ATR FT-IR imaging represents an additional tool to determine molecular orientation.

## Electronic supplementary material

Supplementary Information

## References

[1] Yang J (2013). The role of satellite remote sensing in climate change studies. Nature Climate Change.

[2] Lu R (2016). High-sensitivity infrared attenuated total reflectance sensors for *in situ* multicomponent detection of volatile organic compounds in water. Nature Protocols.

[3] Stanley R (2012). Plasmonics in the mid-infrared. Nature Photonics.

[4] Wegst UGK, Bai H, Saiz E, Tomsia AP, Ritchie RO (2015). Bioinspired structural materials. Nature Materials.

[5] Schliesser A, Picqué N, Hänsch TW (2012). Mid-infrared frequency combs. Nature Photonics.

[6] Yoshioka T, Tashiro K, Ohta N (2016). Molecular orientation enhancement of silk by the hot-stretching-induced transition from a-helix-HFIP complex to *β*-sheet. Biomacromolecules.

[7] Tao H, Kaplan DL, Omenetto FG (2012). Silk materials: A road to sustainable high technology. Adv. Mater..

[8] Li G (2015). Silk-based biomaterials in biomedical textiles and fiber-based implants. *Adv. Healthcare*. Materials.

[9] Liu, X. & Zhang, K.-Q. Silk Fiber - Molecular Formation Mechanism, Structure- Property Relationship and Advanced Applications (Intech, 2014).

[10] Qin N (2016). Nanoscale probing of electron-regulated structural transitions in silk proteins by near-field IR imaging and nano-spectroscopy. Nature Communications.

[11] Cebe P (2013). Beating the heat - fast scanning melts silk beta sheet crystals. Macromolecules.

[12] Maximova K (2016). Silk patterns made by direct femtosecond laser writing. Biomicrofluidics.

[13] Asakura, T. & Miller, T. (eds) *Biotechnology of silk*. Biologically-inspired systems 5 (Springer Science + Business Media, Dordrecht), (2014).

[14] Balčytis A (2017). Silk: Optical properties over 12.6 octaves THz-IR-Visible-UV range. Materials.

[15] Shao Z, Vollrath F (2002). Surprising strength of silkworm silk. Nature.

[16] Du N (2006). Design of superior spider silk: From nanostructure to mechanical properties. Biophysical J..

[17] Dalle-Ferrier C (2016). Why many polymers are so fragile: A new perspective. J. Chem. Phys..

[18] Riekel C (1999). Aspects of X-ray diffraction on single spider fibers. Int. J. Biological Macromolecules.

[19] Sampath S, Yarger JL (2015). Structural hysteresis in dragline spider silks induced by supercontraction: an X-ray fiber micro-diffraction study. RSC Advances.

[20] Boulet-Audet, M., Lefèvre, T., Buffeteau, T. & Pézolet, M. Attenuated total reflection infrared spectroscopy: An efficient technique to quantitatively determine the orientation and conformation of proteins in single silk fibers **62**, 956–962 (2008). 10.1366/000370208785793380 18801233

[21] Boulet-Audet M, Buffeteau T, Boudreault S, Daugey N, Pézolet M (2010). Quantitative determination of band distortions in diamond attenuated total reflectance infrared spectra. J. Phys. Chem. B.

[22] Paquet-Mercier F, Lefèvre T, Augera M, Pézolet M (2013). Evidence by infrared spectroscopy of the presence of two types of *β*-sheets in major ampullate spider silk and silkworm silk. Soft Matter.

[23] Hernández Cruz D, Rousseau M-E, West MM, Pézolet M, Hitchcock AP (2006). Quantitative mapping of the orientation of fibroin *β*-sheets in B. mori cocoon fibers by scanning transmission X-ray microscopy. Biomacromolecules.

[24] Rousseau M-E, Hernández Cruz D, Hitchcock MMWAP, Pézolet M (2007). Nephila clavipes spider dragline silk microstructure studied by scanning transmission X-ray microscopy. J. Am. Chem. Soc..

[25] Huang X, Liu G, Wang X (2012). New secrets of spider silk: Exceptionally high thermal conductivity and its abnormal change under stretching. Adv. Mater..

[26] Martel A, Burghammer M, Davies RJ, Riekel C (2007). Thermal behavior of bombyx mori silk: Evolution of crystalline parameters, molecular structure, and mechanical properties. Biomacromolecules.

[27] Zhang L, Bai Z, Ban H, Liu L (2015). Effects of the amino acid sequence on thermal conduction through *β*-sheet crystals of natural silk protein. Phys. Chem. Chem. Phys..

[28] Zhang L, Chen T, Ban H, Liu L (2014). Hydrogen bonding-assisted thermal conduction in *β*-sheet crystals of spider silk protein. Nanoscale.

[29] Papadopoulos P, Sölter J, Kremer F (2007). Structure-property relationships in major ampullate spider silk as deduced from polarized FTIR spectroscopy. J. European Physical E.

[30] Papadopoulos P, Sölter J, Kremer F (2009). Hierarchies in the structural organization of spider silk - a quantitative model. Colloid Plymer Sci..

[31] Ene R, Papadopoulos P, Kremer F (2009). Combined structural model of spider dragline silk. Soft Matter.

[32] Hikima Y, Morikawa J, Hashimoto T (2011). FT-IR image processing algorithms for in-plane orientation function and azimuth angle of uniaxially drawn polyethylene composite film. Macromolecules.

[33] Vongsvivut J (2014). Rapid determination of protein contents in microencapsulated fish oil supplements by ATR-FTIR spectroscopy and partial least square regression (PLSR) analysis. Food Bioprocess Technol..

[34] Taddei P, Monti P (2005). Vibrational infrared conformational studies of model peptides representing the semicrystalline domains of bombyx mori silk fibroin. Biopolymers.

[35] Lu Q (2010). Water-insoluble silk films with silk i structure. Acta Biomater..

[36] Rousseau M-E, Lefevre T, Beaulieu L, Asakura T, Pezolet M (2004). Study of protein conformation and orientation in silkworm and spider silk fibers using Raman microspectroscopy. Biomacromolecules.

[37] Hikima Y, Morikawa J, Hashimoto T (2012). Imaging of two-dimensional distribution of molecular orientation in poly(ethylene oxide) spherulite using IR spectrum and birefringence. Macromolecules.

[38] Ling S, Qi Z, Knight DP, Shao Z, Chen X (2011). Synchrotron FTIR microspectroscopy of single natural silk fibers. Biomacromolecules.

[39] Alberts, B. *et al*. *Molecular Biology of the Cell*, chap. 3. 2002, 4 edn (New York, Garland Science, 2002).

[40] Haynes, W. M. (ed.) *CRC Handbook of Chemistry and Physics*, 94 edn (CRC Press, Boca Raton, 2016).

[41] Malinauskas M (2016). Ultrafast laser processing of materials: from science to industry. Light: Sci. Appl..

[42] Tsuboi Y, Goto M, Itaya A (2001). Pulsed laser deposition of silk protein: Effect of photosensitized-ablation on the secondary structure in thin deposited films. J. Appl. Phys..

[43] Tsuboi Y, Ikejiri T, Shiga S, Yamada K, Itaya A (2001). Light can transform the secondary structure of silk protein. Appl. Phys. A.

[44] Teramoto H, Miyazawa M (2005). Molecular orientation behavior of silk sericin film as revealed by ATR infrared spectroscopy. Biomacromolecules.

[45] Li C (2016). Regenerated silk materials for functionalized silk orthopedic devices by mimicking natural processing. Biomaterials.

[46] Cunningham A, Davis GR, Ward IM (1974). Determination of molecular orientation by polarized infra-red radiation in an oriented polymer of high polarizability. Polymer.

[47] Cunningham A, Ward IM, Wills H, Zichy V (1974). An infra-red spectroscopic study of molecular orientation and conformational changes in poly(ethylene terephthalate). Polymer.

[48] Jarvis DA, Hutchinson IH, Bower DI, Ward IM (1980). Characterization of biaxial orientation in poly(ethylene terephthalate) by means of refractive index measurements and raman and infra-red spectroscopies. Polymer.

[49] Bieri M, Burgi T (2005). Adsorption kinetics, orientation, and self-assembling of n-acetyl-l-cysteine on gold: A combined ATR-IR, PM-IRRAS, and QCM study. Phys. Chem. B.

