# Author Correction: Orientational Mapping Augmented Sub-Wavelength Hyper-Spectral Imaging of Silk

**DOI:** 10.1038/s41598-018-19721-3

**Published:** 2018-01-24

**Authors:** Meguya Ryu, Armandas Balčytis, Xuewen Wang, Jitraporn Vongsvivut, Yuta Hikima, Jingliang Li, Mark J. Tobin, Saulius Juodkazis, Junko Morikawa

**Affiliations:** 1 Tokyo Institute of Technology, Meguro-ku Tokyo, 152-8550 Japan; 2 Nanotechnology facility, Center for Micro-Photonics, Swinburne University of Technology, John st., Hawthorn, Victoria 3122 Australia; 3 Department of Laser Technologies, Center for Physical Sciences and Technology, Savanoriu Ave. 231, LT-02300 Vilnius, Lithuania; 4 Infrared Microspectroscopy Beamline, Australian Synchrotron, Clayton, Victoria 3168 Australia; 5 Department of Chemical Engineering, Graduate School of Engineering, Kyoto University, Nishikyo-ku, Kyoto 615-8510 Japan; 6 Institute for Frontier Materials, Deakin University, Waurn Ponds, Victoria 3217 Australia; 7 Center of Nanotechnology, King Abdulaziz University, Jeddah, 21589 Saudi Arabia; 8 Melbourne Center for Nanofabrication, Australian National Fabrication Facility, Clayton, 3168 Australia

Correction to: *Scientific Reports* 10.1038/s41598-017-07502-3, published online 07 August 2017

In this Article, Junko Morikawa is incorrectly listed as being affiliated with ‘Infrared Microspectroscopy Beamline, Australian Synchrotron, Clayton, Victoria, 3168, Australia’

The correct affiliation is listed below:

‘Tokyo Institute of Technology, Meguro-ku, Tokyo, 152-8550, Japan’

This error has now been corrected in the PDF and HTML versions of the paper, and in the accompanying Supplementary Information.

