# Supplementary material for: Orientational Mapping Augmented Sub-Wavelength Hyper-Spectral Imaging of Silk
Source: Sci Rep. 2017 Aug 7;7:7419. doi: 10.1038/s41598-017-07502-3 (PMC5547124; doi:10.1038/s41598-017-07502-3)
Supplement: Supplementary file 1 — Supplementary Information [file 41598_2017_7502_MOESM1_ESM.pdf]

Supplement: Orientational Mapping Augmented Sub-Wavelength Hyper-Spectral Imaging of Silk

Meguya Ryu[1], Armandas Balčytis[2,3], Xuewen Wang[2], Jitraporn Vongsvivut[4], Yuta Hikima[5], Jingliang Li[6], Mark J. Tobin[4], Saulius Juodkazis[2,7,8*], and Junko Morikawa[1,*]

[1]Tokyo Institute of Technology, Meguro-ku, Tokyo 152-8550, Japan
[2]Nanotechnology facility, Center for Micro-Photonics, Swinburne University of Technology, John st., Hawthorn, Victoria 3122, Australia
[3]Department of Laser Technologies, Center for Physical Sciences and Technology, Savanoriu Ave. 231, LT-02300 Vilnius, Lithuania
[4]Infrared Microspectroscopy Beamline, Australian Synchrotron, Clayton, Victoria 3168, Australia
[5]Department of Chemical Engineering, Graduate School of Engineering, Kyoto University, Nishikyo-ku, Kyoto 615-8510, Japan
[6]Institute for Frontier Materials, Deakin University, Waurn Ponds, Victoria 3217, Australia
[7]Center of Nanotechnology, King Abdulaziz University, Jeddah 21589, Saudi Arabia
[8]Melbourne Center for Nanofabrication, Australian National Fabrication Facility, Clayton 3168, Australia
[*]SJ: sjuodkazis@swin.edu.au; JM: morikawa.j.aa@m.titech.ac.jp

ABSTRACT

This is an online supplement. References to figures 1 and 2 are those in this supplement; references to other figures are from the article.

The order parameters and percentage of crystalline phase (β-sheets) in silk determined by different methods strongly varies[1]. To quantify and visualise the order of molecular alignment inside a T-cross-section of silk and to compare with another synchrotron FT-IR absorbance measurement[1], the second order momentum $P_2(\theta)$ was calculated (Eqn. 2) for the orientation angle θ (Fig. 4):

$$P_2(\theta) = \frac{3\langle\cos^2\theta\rangle - 1}{2}.$$

By using two IR absorptions at perpendicular and parallel polarisations in respect to the selected orientation (along silk fiber), the second order parameter P_2 can be written as follows:

$$P_2(\theta) = \frac{A_1 - A_2}{A_1 + 2A_2}, \tag{1}$$

where $\mathbf{n}$ is the direction of selected axis and θ is the angle of the transition dipole moment (Fig. 1). Incident light polarization direction of A_1 is parallel while A_2 is perpendicular to $\mathbf{X}_1$ axis.

Estimation of the polymer chain second order momentum P_2 is carried out considering three angles between the measured transition dipole moment, θ, the chain axis, α_m, and mutual orientation between polarisation and chain, φ, depicted in Fig. 1. The following relations applies[2,3]:

$$P_2(\theta) = P_2(\alpha_m) \times P_2(\varphi). \tag{2}$$

Explicitly,

$$\begin{aligned} P_2(\varphi) = \frac{P_2(\theta)}{P_2(\alpha_m)} &\equiv \frac{A_1 - A_2}{A_1 + 2A_2} \times \frac{2}{3\langle\cos^2\theta\rangle - 1} \\ &= \frac{D-1}{D+2} \times \frac{2}{3\langle\cos^2\theta\rangle - 1}, \end{aligned} \tag{3}$$

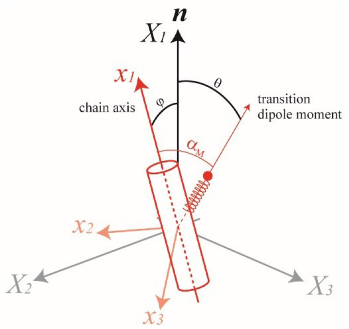

Figure 1. Orientation of the transition dipole in respect to a selected orientation $\mathbf{n} \parallel \mathbf{X}_1$; $X_1Y_1Z_1$ coordinate system. The orientation of a polymeric chain is given by direction $\mathbf{x}_1$ in the coordinate system $x_1y_1z_1$ where α_m defines orientation of the dipole. The angle φ defines tilt between the two coordinate systems.

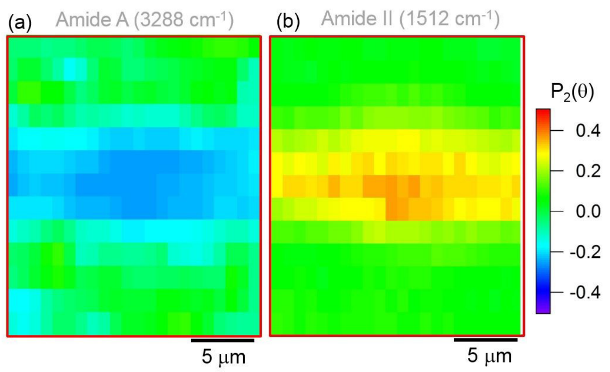

Figure 2. The $P_2(\theta)$ order parameter distribution along silk fiber for different transition dipole moments of Amide A and Amide II bands, which are perpendicular (see, the difference in color map).

where $D = A_{0^\circ}/A_{90^\circ} \equiv A(\varphi_1)/A(\varphi_3)$ is the ratio of the absorbances.

Here, we first estimate the second order parameter P_2 of the measured transition dipole moment which can be directly compared[4] with the value reported in the polarized Raman scattering[5]:

$$P_2(\theta) = \frac{D-1}{D+2}. \quad (4)$$

The strong orientation of C-N, C=O, and N-H present in β-sheets can now be clearly revealed by the direct measurement and polarization analysis from the volumes with cross-sections smaller or comparable with the wavelength; this was not accessible earlier[1]. The second order parameter, $P_2(\theta)$ is plotted in Fig. 2 for the two different transition dipole moments showing a prevalent molecular ordering in the fiber. The molecular alignment in (a) is mainly perpendicular to the horizontal direction, hence, the value of $P_2(\theta) < 0$ is negative while $P_2(\theta) > 0$ (b) for the transition dipole moment aligned mainly perpendicularly. The epoxy region surrounding the fiber shows order parameter $P_2(\theta) = 0$ as expected for the random molecular alignment.

For the further evaluation of the second order momentum P_2 of the chain axis (Fig. 1), the value of α_m is required. If α_m of the Amide A and Amide I is assumed 90°, then, α_m of the Amide II can be calculated (Eqn. 6) as 27.6° according to the previous simulation[6]. The $P_2(\varphi)$ can be also regarded as an in-plane distribution.

Figure 4 is the spatial distribution of absorbance calculated for the Amide A, Amide I and Amide II by Eqns. 1-2 with estimation of $P_2(\theta)$ and $P_2(\varphi)$ summarized in Table 1. The strongest alignment was observed for the C=O bonds which are participating in the hydrogen bonded β-sheets -NH$\cdots$O=C- with $P_2(\theta) = -0.29 \pm 0.026$ (uncertainty has been evaluated from three neighboring pixels along the fiber). This is comparable with $P_2(\theta) = -0.36$ obtained in Raman scattering from silk fibers[5]; scattering and diffraction anisotropy of the fiber has an affect onto measurements while flat samples of the T cross sections were measured in our study. Microtome slices might introduce an orientation artefact on the surfaces, however, this is expected to be of a secondary importance.

Table 1. The second moment P_2 of the orientation function (Herman's function [-0.5 to 1]) for the Amide bands at wavenumbers λ; a negative polariser angle is counted clockwise.

Amide	λ	Absorbance:		D	$P_2(\theta)$	α_m	$P_2(\varphi)$
	(cm^{-1})	0° –	90° |	A_{0°/A_{90°		deg.[6]	
A N-H	3290	0.44	1.12	0.39±0.04	-0.22±0.026	90	0.51±0.05
I C=O	1624	0.33	1.05	0.31±0.03	-0.29±0.026	90	0.59±0.06
II C-N	1510	1.63	0.61	2.67±0.3	0.28±0.042	27.6	0.53±0.06

References

1. Ling, S., Qi, Z., Knight, D. P., Shao, Z. & Chen, X. Synchrotron FTIR microspectroscopy of single natural silk fibers. *Biomacromolecules* **12**, 3344–3349 (2011).
2. Cunningham, A., Davis, G. R. & Ward, I. M. Determination of molecular orientation by polarized infra-red radiation in an oriented polymer of high polarizability. *Polymer* **15**, 743–748 (1974).
3. Cunningham, A., Ward, I. M., Wills, H. & Zichy, V. An infra-red spectroscopic study of molecular orientation and conformational changes in poly(ethylene terephthalate). *Polymer* **15**, 749–756 (1974).
4. Jarvis, D. A., Hutchinson, I. H., Bower, D. I. & Ward, I. M. Characterization of biaxial orientation in poly(ethylene terephthalate) by means of refractive index measurements and raman and infra-red spectroscopies. *Polymer* **21**, 41 – 54 (1980).
5. Rousseau, M.-E., Lefevre, T., Beaulieu, L., Asakura, T. & Pezolet, M. Study of protein conformation and orientation in silkworm and spider silk fibers using Raman microspectroscopy. *Biomacromolecules* **5**, 2247 – 2257 (2004).
6. Bieri, M. & Burgi, T. Adsorption kinetics, orientation, and self-assembling of n-acetyl-l-cysteine on gold: A combined ATR-IR, PM-IRRAS, and QCM study. *Phys. Chem. B* **109**, 22476 – 22485 (2005).
